# Smart-Sensor for the Automatic Detection of Electromechanical Faults in Induction Motors Based on the Transient Stray Flux Analysis

**DOI:** 10.3390/s20051477

**Published:** 2020-03-08

**Authors:** Israel Zamudio-Ramírez, Roque Alfredo Osornio-Ríos, Jose Alfonso Antonino-Daviu, Alfredo Quijano-Lopez

**Affiliations:** 1 Engineering Faculty, San Juan del Río Campus, Universidad Autónoma de Querétaro, Av. Río Moctezuma 249, San Juan del Río, Querétaro 76807, Mexico; isra.zam.ram@hotmail.com (I.Z.-R.); raosornio@hspdigital.org (R.A.O.-R.); 2 Instituto Tecnológico de la Energía, Universitat Politècnica de València (UPV), Camino de Vera s/n, 46022 Valencia, Spain; aquijano@ite.upv.es

**Keywords:** induction motor, smart-sensor, triaxial stray flux sensor, time–frequency transforms

## Abstract

Induction motors are essential and widely used components in many industrial processes. Although these machines are very robust, they are prone to fail. Nowadays, it is a paramount task to obtain a reliable and accurate diagnosis of the electric motor health, so that a subsequent reduction of the required time and repairing costs can be achieved. The most common approaches to accomplish this task are based on the analysis of currents, which has some well-known drawbacks that may lead to false diagnosis. With the new developments in the technology of the sensors and signal processing field, the possibility of combining the information obtained from the analysis of different magnitudes should be explored, in order to achieve more reliable diagnostic conclusions, before the fault can develop into an irreversible damage. This paper proposes a smart-sensor that explores the weighted analysis of the axial, radial, and combination of both stray fluxes captured by a low-cost, easy setup, non-invasive, and compact triaxial stray flux sensor during the start-up transient through the short time Fourier transform (STFT) and characterizes specific patterns appearing on them using statistical parameters that feed a feature reduction linear discriminant analysis (LDA) and then a feed-forward neural network (FFNN) for classification purposes, opening the possibility of offering an on-site automatic fault diagnosis scheme. The obtained results show that the proposed smart-sensor is efficient for monitoring and diagnosing early induction motor electromechanical faults. This is validated with a laboratory induction motor test bench for individual and combined broken rotor bars and misalignment faults.

## 1. Introduction

Electric motors are very important devices for many industrial processes, as they are widely used as primary movers of most of the loads involved in those applications. Their vast usage can represent, in terms of electrical consumption, between 40% and 60% of the total in any industrial site [1]. Induction motors are especially widespread owing to their robustness, easy maintenance, low cost, and versatility [2]. However, regardless of their great advantages and exceptional features, these machines are susceptible to failure during their service life situations that compromise their performance and reliability, bringing time-gradually faults, and efficiency losses, which in turn, if not attended at incipient stages, can lead to the shutdown of processes, causing huge time and economical losses. In this regard, new techniques and methodologies to detect failures in electric motors have been proposed, where the appearance of new sensors in combination with optimal signal processing techniques have marked a trend that turns into timely diagnoses with greater reliability.

In recent years, the evaluation and diagnosis of the healthiness state of electric motors through several monitoring techniques and strategies has become a task of great relevance, as a timely maintenance through an early detection of irregularities during normal machine operation can be achieved. For such purposes, a wide variety of approaches has been developed. Most of them use the information obtained from various physical quantities captured by basic primary sensors during normal motor operation. In this context, the analysis of conventional physical magnitudes such as vibration signals, infrared data, partial discharges, ultrasounds, and currents, among others, has been proposed [3]. Each of these techniques is subjected to its own advantages and disadvantages. In this regard, the motor current signature analysis (MCSA) approach has become an extensively used method over the last decades, as several faults can be diagnosed with this approach [4], with frequency-domain procedures being the most preferable ones, as they can deliver results with high reliability [5,6]. However, this approach has some well-known drawbacks when analyzing faults in certain circumstances; for instance, they are not immune to false indications caused by the presence of load torque oscillations or supply voltage fluctuations, because specific spectral components that are non-related to electromechanical faults may appear at same locations as those related to electromechanical faults [7]. Some of the above-mentioned drawbacks have been enlisted and discussed by some investigations [8,9,10]. As a result of these particularities, the reliability of the final diagnosis can be deteriorated, causing false indications, a situation which has led the researchers to explore alternative sources of information to overcome some of the aforementioned problems.

In order to find alternatives that can provide a complementary diagnosis to well-established techniques, emergent and modern physical magnitudes, such as the stray flux data analysis, have been investigated for induction motor (IM) fault diagnosis purposes. For instance, in [11], intern-turn short circuits are effectively diagnosed by identifying peaks of greater amplitude at specific frequencies in the Fourier spectrum of the axial, radial, and combination of both stray fluxes captured by coil-based sensors distributed on the frame of the machine at specific positions. In a similar way, in [12,13,14,15,16], individual faults such as static and dynamic rotor eccentricities, bearing faults, and even the insulation ageing of the stator windings are adequately identified by means of the stray flux analysis. However, as most of these techniques base the final diagnosis on specific peaks amplified by the presence of a failure, the reliability of the same can be reduced because, for low load conditions, there is usually an overlap between the amplified harmonics caused by the failure and the fundamental frequency [17]. To diminish the effects of this particularity, modern methodologies use more sophisticated techniques suitable for transient analysis during machine transient startup, in which very specific patterns can be located in the different axial and radial components of the stray flux. Thus, several investigations have demonstrated the high effectiveness achieved when using the transient analysis of stray flux to diagnose several electromechanical failures in electric motors [18,19,20,21,22,23,24].

In this context, it is noteworthy, on the one hand, that the final diagnosis in some of these works is based in the analysis of just one stray flux component (either axial or radial), however, it is desirable to obtain the both components individually at the same time, as some failures follow to have an axial nature, while others show a radial origin [25]. Furthermore, in some of these works, the need to distribute more than one sensor in order to obtain the axial and radial components of the stray flux and reliably diagnose some faults is unavoidable, and even so, some proposed sensors demand considerable space on the frame of the motor. Nevertheless, practically, it is not always possible to have access to the whole frame of the machine. In this regard, some investigations, such as in [26], have been particularly dedicated to studying the impact of diverse stray flux sensors for the condition monitoring of electrical machines, making evident the relevance of the primary sensors used for this task. On the other hand, in order to carry out a final diagnosis in most of the proposed methodologies, it is required to analyze the captured signals on a personal computer, which is a non-optimum solution for the current necessities and demands, where the constant monitoring and diagnosis of the machine is of paramount concern in order to avoid potential motor outages and production downtimes. With that, there has been a remarkable trend to combine/modify and improve these techniques with new sensor technologies in order to provide a portable system able to perform on-site diagnosis; in this way, so-called smart-sensors are appearing, where the information captured by one or more primary sensors is processed in a main processing unit that complies with certain functionalities like processing, communication, and integration. Smart-sensors have been used for different applications such as the identification of broken rotor bars and unbalance in induction motors using current signals [27,28], real-time high-resolution frequency measurement [29], and monitoring and diagnosis of faults in distinct industrial applications [30,31], among others. Evidently, it is highly desirable to automatically diagnose induction motor faults through innovative methodologies using the information provided by physical magnitudes highly related to the fault. As has been demonstrated, the study of the stray flux has been shown to be an excellent alternative and complementary approach to conventional methods for the diagnostic of electric motor faults, as certain drawbacks can be overcome [9]. In order to acquire such signals, it is very important to obtain the axial and radial stray flux components at the same time from any point on the frame, as higher reliable results can be achieved.

This work introduces a smart-sensor based on primary hall effect sensors, which is able to capture the stray flux in different directions in its axial, radial, and combination of both components (by installing it in just one position on the frame). The main processing unit of the proposal consists of a set of mathematical time–frequency decomposition (TFD), filtering, and classification tools. For the purpose of this work, the main module relies on the analysis of the radial and axial stray fluxes under transient by applying the short time Fourier transform (STFT). Furthermore, in order to automatically establish a final diagnosis, the main processing unit uses statistical parameters of the transient to feed a linear discriminant analysis dimensionality reduction (LDA) and then a feedforward neural network (FFNN), which classifies the healthiness state of the analyzed induction motor. The proposed smart-sensor is validated under a laboratory induction motor test bench for combining one broken rotor bar, two broken rotor bars, and misalignment. The results prove the capabilities of the proposed prototype.

## 2. Materials and Methods

### 2.1. Stray Flux Analysis

**Figure 1:**
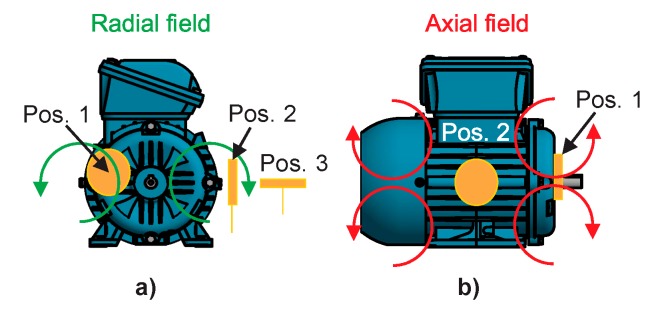
Magnetic stray flux components: (**a**) radial stray flux; (**b**) axial stray flux.

According to the authors of [32], the stray flux is the magnetic flux that radiates outside the frame of the machine induced by stator and rotor currents. These currents (and hence the stray flux) are affected when the electric motor operates under a fault condition. As reported by the authors of [33], the stray flux can be analyzed through its two magnetic components: axial and radial stray flux. It is known that the axial field is generated by currents in the stator end windings or rotor cage end ring and is located in a plane that comprises the machine axis, while the radial field is located in a plane perpendicular to the machine axis. In agreement with the authors of [11], it is possible to separately capture the axial and radial stray flux components and even the combination of both by installing suitable sensors on the frame of the motor at specific positions. Figure 1 shows the presumed circulation of the field lines of the axial and radial stray fluxes and the alternative positions in which coil-based sensors are installed to capture them. The axial field can be measured by position 1 and the radial field through position 3, while position 2 captures the combination of both stray fluxes.

#### Broken Rotor Bars and Misalignment Effects on the Stray Flux

Several investigations have shown that the stray flux is modified when the electric motor is operating under a fault. As has been pointed out in those works, when the motor presents rotor damages, several components are induced in the Fourier spectrum of the stray flux signals at steady-state regime, with the most significant being the following ones:

- Components of axial nature that can also be amplified by the presence of eccentricities and/or misalignments in the machine [34,35]. These components are observed at specific harmonics according to Equations (1) and (2).
  (1) $$f_{MAL}=s\cdot f\;$$
  (2) $$f_{MAL2}=3\cdot s\cdot f\;$$
  where *s* = slip and *f* = supply frequency.
- Sideband harmonics appearing in the radial stray flux component that are also seen in the spectrum of the steady-state current [22,32]. These components follow Equation (3).
  (3) $$f_{LSH}=f\cdot \left(1\pm 2\cdot s\right)$$

On the other hand, in the case of mixed eccentricities or misalignments. The amplified harmonics can be observed at frequencies according to Equation (4) [36].

(4) $$f_{ecc}=f\cdot \left(1\pm m\left(1-s\right)/p\right)$$

where *m* = 1,2… and *p* = number of pole pairs.

**Figure 2:**
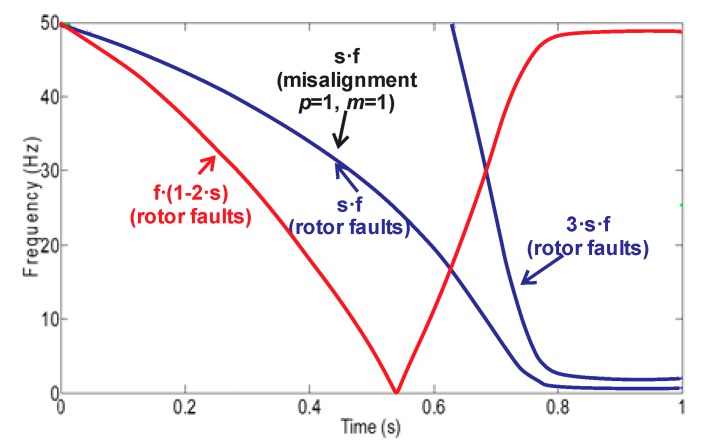
Theoretical evolutions under the starting for the broken bar–related components (axial and radial) and for the misalignment components.

Equations (1)–(4) suggest that the harmonics related to the fault depend on the motor slip, so that it is possible to observe their development during the transient startup, where the slip starts at a maximum value when the motor is not running, and reaches a minimum value in the steady state. According to Equations (1)–(4), the theoretical patterns expected during the transient start-up in the case of broken rotor bars and misalignments can be seen in Figure 2.

### 2.2. Artificial Neural Network

**Figure 3:**
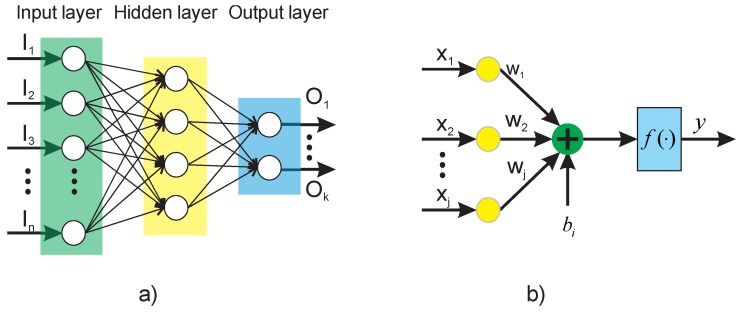
Artificial neural network: (**a**) feed–forward neural network (FFNN) architecture; (**b**) functional structure of a neuron.

Artificial neural networks (ANNs) are computational models capable of solving classification and pattern recognition problems through complete algorithmic structures [37]. Among the most common ANN architectures, feed-forward neural networks (FFNN) have been widely used as this model is very simple and practical, and in computational terms, its calculation represents a very low burden. Moreover, this neural network structure facilitates the possibility of generating automated results by generalizing suitably about the data with which it is trained. FFNN have found applications on solving classification problems and approximating real-valued functions. The most general structure of FFNN is composed by a layered architecture having essentially one input layer, one or more hidden layers, and one output layer, as shown on Figure 3a, where *I_i_* are the inputs of the FFNN and *O_i_* correspond to the outputs of the model. Each layer has one or more elementary units called neurons, whose processing capability is stored in the connections by synaptic weights, and whose adaptation depends on learning [38] (see Figure 3b). The mathematical model of each neuron is given by Equation (5), where *y* is the output of the neuron, *w_i_* are the synaptic weights, *x_i_* are the inputs of the neuron, *b* is the bias, *f*(*·*) is the activation function, and *n* is the total number of inputs.

(5) $$y=f\left(\sum_{i=1}^{n}w_{i}x_{i}+b\right)$$

### 2.3. Linear Discriminant Analysis

The linear discriminant analysis (LDA) is a supervised dimensionality reduction technique that is generally used for classification purposes. The goal of this classifier is to project the dataset of *d*-dimensional vectors onto a smaller subspace *s* (where *s ≤ d*) by maximizing the linear separability between data of different classes finding a linear mapping [39]. The mathematical procedure to perform LDA is described in summary below and can be found in detail in [40]:

1. Compute *d*-dimensional mean vectors of the input matrix.
2. Compute between-class matrix and within-class scatter matrix.
3. Compute eigenvectors and eigenvalues.
4. Select linear discriminants for the new feature subspace and form an eigenvector matrix.
5. Use the eigenvector matrix to transform the vectors onto the new lower dimensional space. Maximize the between-class matrix and minimize within-class scatter matrix.

### 2.4. Short Time Fourier Transform (STFT)

The STFT is a signal processing technique that allows the decomposition of a time domain signal into its time–frequency components by getting the frequency content enclosed on local sections through detaching them into time windows, where each window is analyzed using the fast Fouier transform (FFT).

Mathematically, STFT is represented by Equation (6):

(6) $$X_{STFT}[m,k]=\sum_{n=0}^{N-1}x[n]g[mL-n]e^{\left(-j\left(2\pi kn/N\right)\right)}$$

where *x*[*n*] is the discrete-time signal; *n* is the time domain index, where *k* = 0,…,*N* – 1, *m* = 0,…,[(*N*/*L*) – 1]; *g* is the windowing function; and *L* determines the time separation among adjacent sections.

### 2.5. Smart-Sensor General Structure

**Figure 4:**
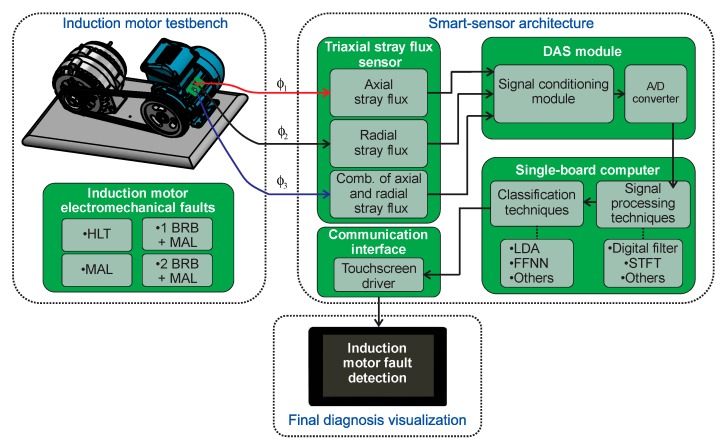
Block diagram of the proposed smart–sensor. BRB + MAL, broken rotor bar + misalignment; HLT, healthy motor; DAS, data acquisition system; A/D, analog/digital; STFT, short time Fourier transform; LDA, linear discriminant analysis; FFNN, feed–forward neural network.

**Figure 5:**
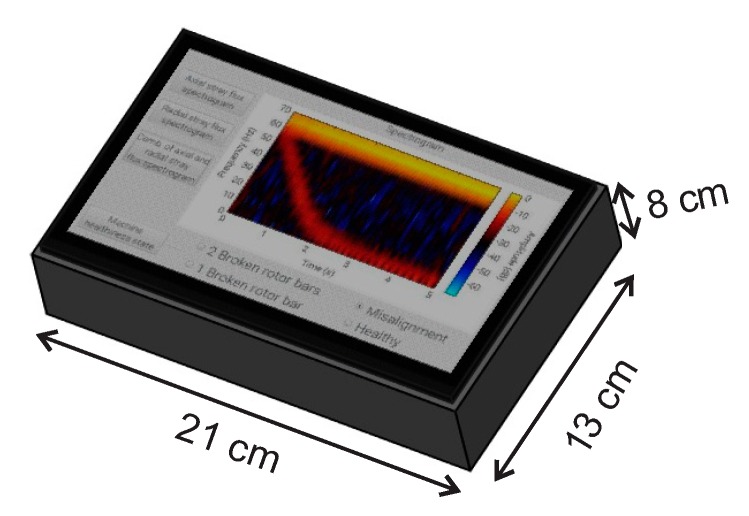
Smart–sensor dimensions.

The proposed smart-sensor is based on a small single-board computer (SBC) and is able to automatically on-site diagnose individual and combined induction motor faults. The diagram of Figure 4 shows a schematic view comprising the structure of the proposal. The dimensions of the proposed smart-sensor can be found in Figure 5. The average total energy consumption of the proposal is 800 mA, and the characteristics of each submodule that make up the smart-sensor are addressed in the following subsections. For the purpose of demonstrating the functionality of the smart-sensor, the healthy motor (HLT), misalignment fault (MAL), and its combination with broken rotor bars are analyzed: one broken rotor bar + misalignment (1 BRB + MAL), and two broken rotor bars + misalignment (2 BRB + MAL), although its use for the diagnosis of other faults and the programming of additional algorithms is not restricted. The main sensor of the system was designed and implemented in our laboratory and is composed of three hall effect primary sensors arranged on a perpendicular axis to each other configuring the so-called triaxial stray flux sensor, which is able to capture the axial and radial stray flux components regardless of its location on the frame of the analyzed motor. The axial (Ø_1_), radial (Ø_2_), and the combination of the axial and radial (Ø_3_) stray flux signals coming from the triaxial sensor are acquired by the DAS (data acquisition system) module, which is essentially composed by a signal condition stage performed through operational amplifiers and a digital to analog converter (ADC). Then, the signal processing is achieved in the SBC by applying suitable time–frequency decomposition techniques. After that, the time–frequency maps obtained are characterized through statistical parameters, which are fed into a classification process unit that uses appropriate supervised machine learning methods in order to automate the diagnosis. Finally, the healthiness state of the machine is showed to the final user through a touchscreen device.

#### 2.5.1. Triaxial Stray Flux Sensor

**Figure 6:**
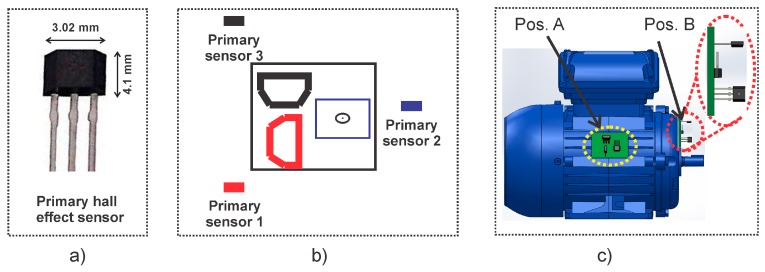
Triaxial stray flux sensor for data acquisition: (**a**) primary hall–effect sensor; (**b**) composition of the triaxial stray flux sensor; (**c**) triaxial stray flux sensor installation.

The main triaxial stray flux sensor is essentially made up of the integration of three individual hall-effect primary sensors, such as the one depicted in Figure 6a. Each of the primary sensors are ALLEGRO ™ microsystems brand, model A1325, having a sensitivity of 5 mV/G. This sensor is 3.02 mm wide by 4.1 mm high, it has an output voltage proportional to the magnetic flux density, low-noise output, wide ambient temperature range: −40 °C to 150 °C, is immune to mechanical stress, and complies with other characteristics as indicated by the manufacturer in their respective data sheet. The primary sensors are located on a perpendicular axis to each other in order to capture the axial, radial, and the combination of the axial and radial stray flux components regardless of their relative location to the motor frame. Figure 6b shows a schematic view of the triaxial stray flux sensor proposed here. The relative positions of the three primary sensors can be observed. In this regard, if the sensors are installed in position A shown in Figure 6c, primary sensor 1 mainly captures the axial flux, primary sensor 2 captures the combination of the axial and radial flux, whereas primary sensor 3 captures the radial flux. Similarly, if the sensors are installed in position B shown in Figure 6c, primary sensor 1 mainly captures the combination of the axial and radial stray flux, primary sensor 2 captures the axial flux, and primary sensor 3 mainly captures the radial stray flux. The main advantage of this sensor is that it can be virtually located anywhere on the frame of the machine in such a way that it still will be able to capture the different stray flux components.

**Figure 7:**
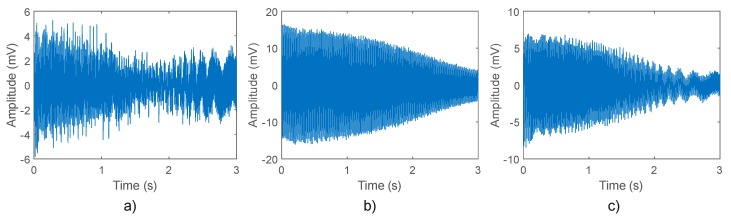
Time–domain signals captured by the proposed triaxial stray flux sensor: (**a**) axial stray flux; (**b**) combination of the axial and radial stray flux; (**c**) radial stray flux.

Figure 7 shows the axial stray flux (in the discrete time-domain) obtained by the triaxial stray flux sensor when installed in the same way as in position A for the case of 2 BRB + MAL, 1 BRB + MAL, and MAL. As can be seen in these signals, it is not obvious to discern the different faults by inspecting them, which suggests the application of relevant signal processing techniques.

#### 2.5.2. Data Acquisition System (DAS Module)

The module developed to adequately acquire the signals from the triaxial stray flux sensor consists of a signal conditioning stage in which an anti-aliasing filter is carried out by means of operational amplifiers, followed by a signal digitalization stage performed through a Texas Instrument microcontroller brand MSP432P401R, which includes an ADC with 14-bit precision, up to 24 input channels, and up to 1 Million samples per second (Msps), which is enough for the purposes of this paper. 

#### 2.5.3. Single-Board Computer-Based Processing Unit

The single-board computer (SBC)-based processor is composed by processing STFT, statistical parameters feature extraction, linear discriminant analysis dimensionality reduction (LDA), and classification FFNN. The SBC processor used in this work is based on a raspberry pi model 3 that has a 4 × ARM Cortex-A53 processor and that bases its operation at a frequency of 1.2 GHz, with an average current consumption of 800 mA in conjunction with the raspberry touchscreen.

**Figure 8:**
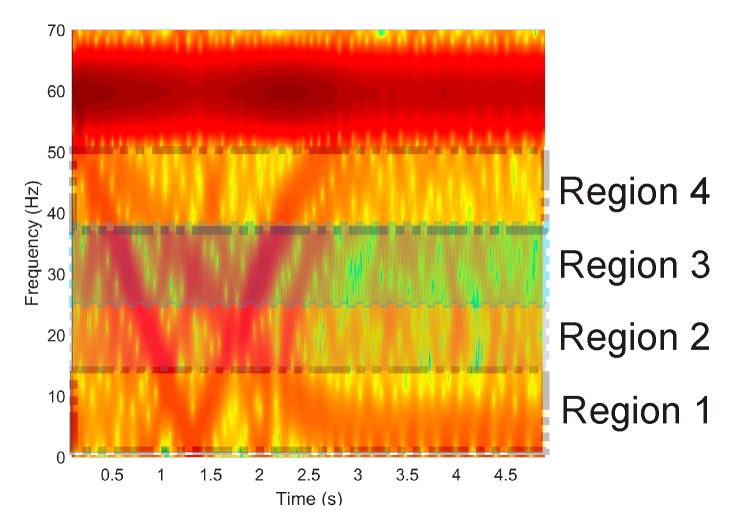
Frequency bands considered in the STFT maps.

In the first stage of the signal processing, the axial, radial, and combination of axial and radial stray fluxes are obtained through the triaxial stray flux sensor. Subsequently, the time–frequency maps of the startup transient are computed using the STFT with a hamming window having a time length of 1024 data points, which, at the sampling frequency used (5 KHz), is 0.2048 s wide, and the hop size used between windows is 256 data points, which represents a 75% overlap, while 1024 points were used for the calculation of the FFT for each window. This gives a frequency resolution of approximately 4.88 Hz and a time resolution of 51.2 ms. These parameters are chosen with the purpose of having a good resolution in time as the transient startup in electric motors is a variable parameter that depends highly on the motor load. Once these maps are obtained, they are subdivided into four regions of interest, which cover a bandwidth below the fundamental power supply component (60 Hz) because, in this region, the majority of harmonics of greater interest (linked with the considered faults) evolve during startup. The following frequency bands or regions were considered: region 1 (~[0–13] Hz), region 2 (~[13–26] Hz), region 3 ((~[26–39] Hz), and region 4 (~[39–52] Hz), as shown in Figure 8.

**Figure 9:**
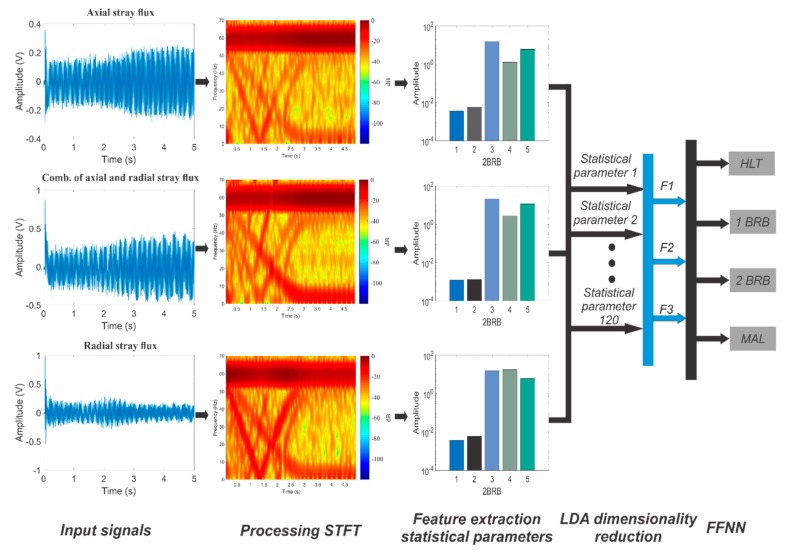
Proposed methodology flow–up.

Once these time–frequency maps are split, several statistical and non-statistical parameters of each of these regions are obtained in order to characterize them. The following ten parameters are considered: (1) signal energy, (2) standard deviation, (3) statistical mean, (4) statistical median, (5) kurtosis, (6) skewness, (7) root mean square, (8) peak-to-average ratio, (9) shape factor, and (10) crest factor. These parameters are chosen because of their ability to provide relevant information about changes and trends in signals by investing a very low cost in computational burden. After those parameters are obtained, an input matrix is generated for the feature reduction through the LDA. After that, the number of input parameters to the LDA is 120 (10 (statistical parameters) by 4 (frequency bands) by 3 (sensors)), which are reduced to three significant features (F1, F2, F3), as this reduction allows to obtain a visual representation of the different fault conditions studied here. Then, the FFNN is trained to separate between classes of the studied failures. This module is firstly trained through the Levenberg–Marquardt algorithm for identifying an HLT condition in the induction motor or the presence of the multiple faults studied here. For this, 40 real sampled signals are carried out under each motor condition, resulting in a total of 160 samples. Of the 40 tests obtained for each case study, 32 of each were used for the training of the FFFN and 8 for validation of the same. This training was performed using specialized software. The FFNN final architecture has 3 inputs (number of parameters after LDA feature reduction), 2 and 10 neurons in the hidden layers, and 4 outputs (one per each condition), which function as indicators of the induction motor condition. The number of 2 and 10 neurons in the hidden layer is selected by trial and error in order to obtain the minimum overall classification error, as suggested in [37]. After training and validation, the final weights and biases of each layer neuron are used for the smart-sensor implementation. The signal flow-up of the proposal is shown in Figure 9.

### 2.6. Experimental Setup

**Figure 10:**
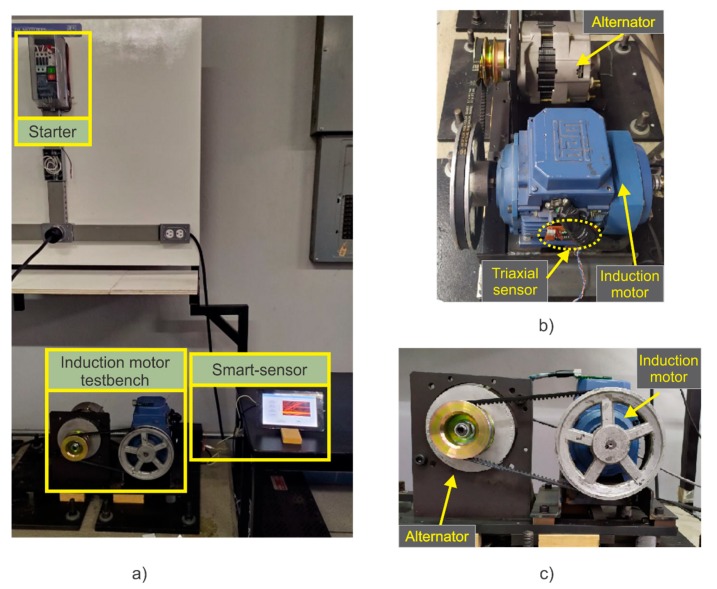
Experimental setup: (**a**) Induction motor testbench, (**b**) side view of the testbench, (**c**) front view of the testbench.

The validation of the proposal is performed by analyzing several signals acquired from an induction motor test bench, in which some of the most common electromechanical faults appearing in this type of motors are studied. The experimental setup used to test the proposed smart-sensor is shown in Figure 10a. The analyzed three-phase induction motor (model WEG 00136APE48T) has two poles, 28 bars, nominal power of 0.74 KW, power factor of 0.87, and a nominal current of 2.9 A. It is fed with 220 Vac at 60 Hz. The mechanical load is provided by an ordinary alternator, which represents 25% of the nominal load for the motor. The stray flux component signals are acquired using the proposed triaxial stray flux sensor placed on the data plate (position A), as shown in Figure 10b and in position B. The DAS uses a sampling frequency of 5000 Hz within a period of 30 seconds, which is enough to capture the startup transient of the motor.

Firstly, the induction motor is tested at healthy conditions in order to establish a reference for the faulty conditions. Then, a very slight mechanical misalignment is induced as this is a very common fault that is usually generated inherently in most mechanical couplings, but that mainly has an impact on wear between the motor and the load. Furthermore, with the purpose of expanding the use and capabilities of the smart-sensor, one and two broken rotor bars were induced and combined with misalignments, allowing the study of two different combined faulty schemes: 1 BRB + MAL and 2 BRB + MAL.

## 3. Results

### 3.1. Study Cases

**Figure 11:**
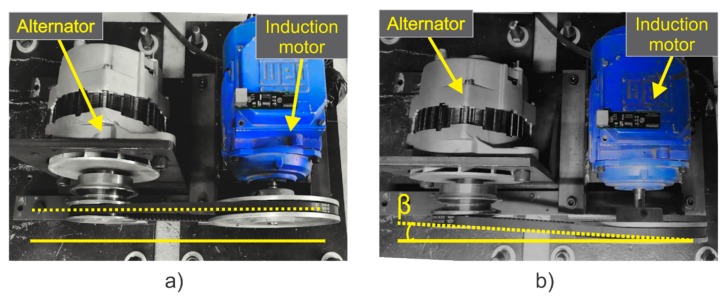
(**a**) Motor aligned; (**b**) motor misaligned.

To demonstrate the satisfactory operation of the smart-sensor proposed here, it was installed in the induction motor test bench, which allowed us to analyze different states of motor health. In order to have a reference in the different cases studied here, the motor was first analyzed in a healthy state. Subsequently, to enable the smart-sensor for the detection of one of the most common mechanical faults, which usually occurs inherently, but which nevertheless produces continuous wear, a very slight misalignment was induced between the motor and the load. The misalignment test was carried out by shifting forward the band in the motor pulley, so that the transverse axes of rotation for the motor and its load were not aligned, forming a gap angle β, as shown in Figure 11b. This condition can be clearly seen by comparing the aligned motor (Figure 11a) and the misaligned motor (Figure 11b).

**Figure 12:**
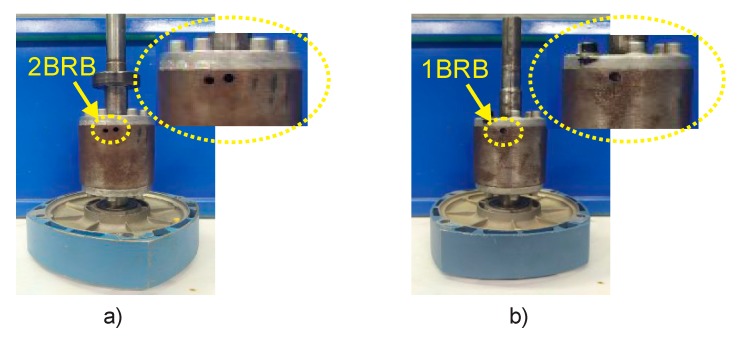
(**a**) Two broken rotor bars; (**b**) one broken rotor bar.

As a second studied fault, broken rotor bars were induced in the motor and combined simultaneously with misalignment. Firstly, the motor was analyzed with two broken rotor bars and misalignment. Subsequently, to demonstrate that faults with a lighter severity can also be diagnosed, one broken rotor bar was induced in combination with misalignment. To produce an artificial broken rotor bar condition, a 2.0 mm diameter hole was drilled in one and two bars of the rotor without harming the rotor shaft. Figure 12a shows the rotor with two broken rotor bars, whereas Figure 12b shows the one broken rotor bar induced in a second rotor, which were used during the test.

### 3.2. Results of the Study Cases Obtained Internally by the Smart-Sensor

**Figure 13:**
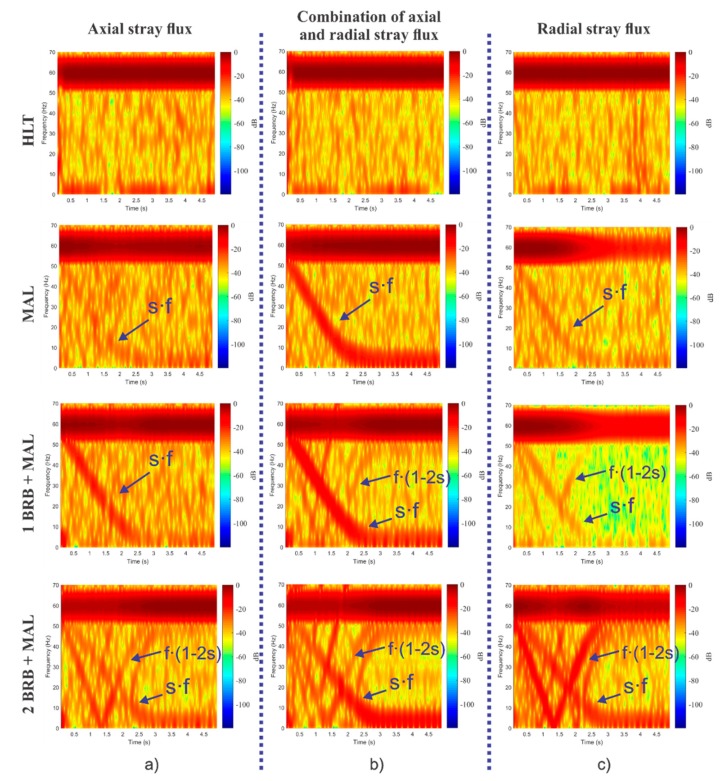
STFT analyses obtained for each one of the induction motor faults studied here when processing the signals provided by the triaxial stray flux sensor when installing it in position A: (**a**) primary sensor 1; (**b**) primary sensor 2; and (**c**) primary sensor 3.

**Figure 14:**
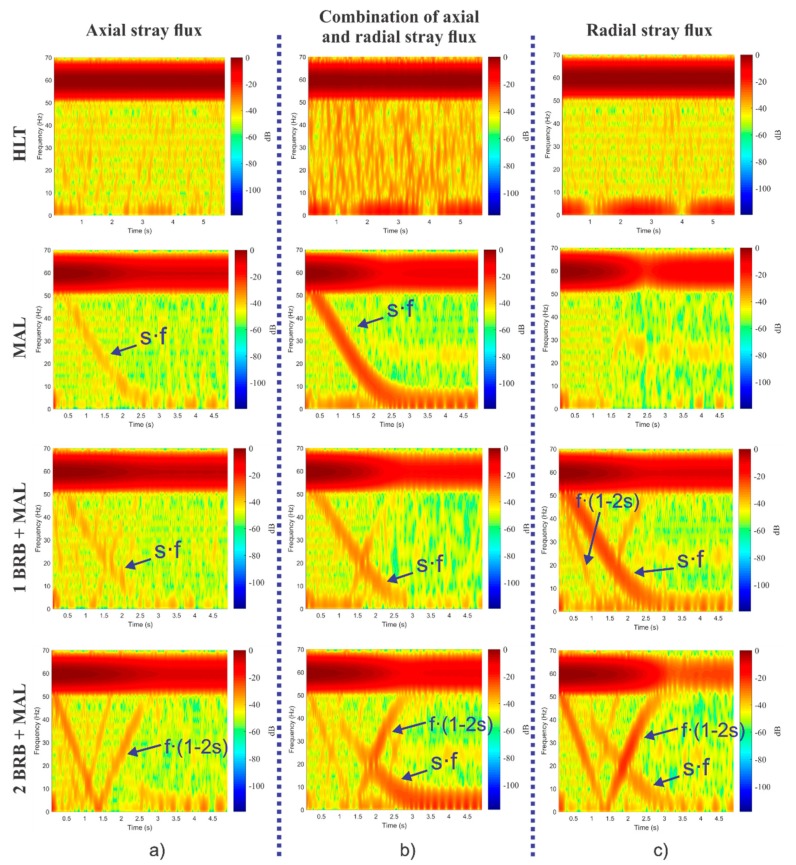
STFT analyses obtained for each one of the induction motor faults studied here when processing the signals provided by the triaxial stray flux sensor when installing it in position A: (**a**) primary sensor 2; (**b**) primary sensor 1; and (**c**) primary sensor 3.

The time–frequency maps obtained internally by the smart-sensor when processing the signals provided by the triaxial stray flux sensor and for each one of the induction motor faults studied here—HLT, MAL, 1 BRB + MAL, and 2 BRB + MAL—are shown in Figure 13 and Figure 14. Figure 13 corresponds to the readings obtained when the sensor is installed in position A shown by Figure 6c, while Figure 14 corresponds to the results obtained when analyzing the signals captured by the triaxial sensor when installed as in position B of Figure 6c. Note the appearance and evolutions of the fault components expected by the theory; on the one hand, the axial component at *s·f* is clearly visible, especially when analyzing the signals provided by primary sensors 1 and 2, which capture the axial and the combination of axial and radial stray fluxes respectively, while the harmonic *f·*(1–2·*s*) is especially visible at primary sensor 3, which mainly captures the radial stray flux.

The performance provided by the smart-sensor (in matters of processing time) when implementing the methodology proposed in this paper is shown in Table 1. To obtain these results, a total of 40 diagnostic tests were run and an average of the processing times was obtained. As shown in Table 1, it takes the smart-sensor 36.9 s to provide a final diagnosis; that is, 30 s for signal acquisition and 6.9 s for signal processing.

**Figure 15:**
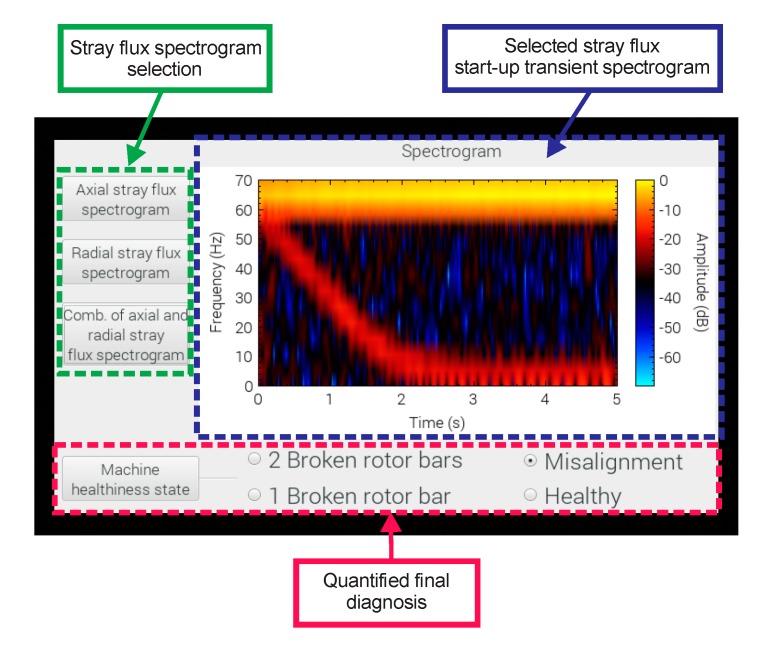
Visualization of the main menu of the smart-sensor.

The interface that shows the results delivered by the smart-sensor to the end user are shown in Figure 15. The interface is divided into three main windows: the one on the left side shows the buttons that select the type of signal to obtain the time–frequency map (axial, radial, or the combination of both stray fluxes); on the right hand side, the spectrogram graph calculated using the STFT of the selected stray flux is located; and in the lower part, the final result obtained through the proposed automated diagnosis is found, which indicates the healthiness state of the machine by selecting from the menu of possible failures to diagnose (HLT, MAL, 1 BRB, and 2 BRB) that are present in the motor under analysis. 

**Figure 16:**
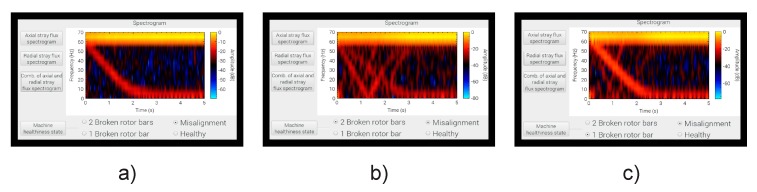
Results delivered by the smart–sensor when tested at different fault conditions: (**a**) misalignment; (**b**) misalignment + two broken rotor bars; (**c**) misalignment + one broken rotor bar.

The final results delivered by the smart sensor to the end user when tested on the induction motor test bench for MAL failure, 2 BRB + MAL, and 1 BRB + MAL can be observed in Figure 16.

Table 2 shows the results delivered by the smart-sensor during induction motor condition identification. A total of 110 tests were executed consecutively for each case study (by installing the smart-sensor) under real machine operating conditions with the purpose of showing the effectiveness of its classification and its capabilities to generate a timely diagnosis on an ongoing basis. The results include the identification of a healthy condition, slight misalignment, and the combination of one and two broken rotor bars with a slight misalignment.

## 4. Discussion

Three different individually and combined fault cases are studied in this work: MAL, 1 BRB + MAL, and 2 BRB + MAL. The results of the automated diagnosis obtained by the smart-sensor show an effectiveness of 100% in the healthy motor and two broken rotor bars combined with misalignment, while the results for one broken rotor bar combined with misalignment and slight misalignment present an effectiveness above 97%. An important characteristic of the proposed smart-sensor is the automatic detection of multiple-combined faults in induction motors with the installation of just one triaxial stray flux sensor, which is in contrast with the reviewed literature, in which the results are obtained considering single faults or multiple-combined faults interpreted offline by an expert. Furthermore, in most of these works, it is necessary to distribute more than one sensor around the frame of the motor to be able to capture both stray flux components separately, which in practical terms is not always feasible.

Through the triaxial stray flux sensor proposed here, it is possible to capture the evolution of induction motor fault harmonics using specialized time–frequency decomposition tools in a concise manner with the advantage of being able to acquire these evolutions from a single position on the motor frame. The time–frequency maps obtained internally by the smart-sensor proposed here (shown in Figure 13 and in Figure 14) demonstrate this fact. In these maps, the appearance of very characteristic patterns can be clearly seen, especially when the motor is operating under a fault condition. On the contrary, when the motor is in a healthy state, no specific pattern is observed. Figure 13a shows the time–frequency maps obtained when analyzing the signal provided by primary sensor 1, which essentially captures the axial stray flux. In these maps, the appearance of the harmonic *s*·*f* (amplified by misalignment faults) and the harmonic *f·*(1–2·*s*) is noticeable, which is amplified by broken rotor bars. However, it is difficult to discern the harmonic *f*·(1–2·*s*) when the motor operates under 1 BRB + MAL. Similarly, Figure 13b shows the time–frequency maps obtained by analyzing the signal captured by primary sensor 2, whose readings correspond to the simultaneous acquisition of the axial and radial stray fluxes. In comparison with the maps obtained through the axial stray flux, the presence of the harmonic *f*·(1–2·*s*) is evident even when the motor operates under 1 BRB + MAL. On the other hand, Figure 13c displays the time–frequency maps obtained by processing the signal provided by primary sensor 3, which essentially corresponds to radial stray flux readings. In these maps, the amplification of the harmonic *f*·(1–2·*s*) with greater intensity (in comparison with the maps obtained through the axial and the combination of axial and radial stray flux) is clear when the motor is operating under 2 BRB + MAL, and even when the motor has 1 BRB + MAL. Furthermore, if the results obtained when analyzing the combination of the axial and radial stray flux for the case of 1 BRB + MAL failure by installing the sensor in position B (see Figure 14) are examined, it is not possible to discern the existence of broken rotor bars, as the component *f*·(1–2·*s*) (characteristic of broken rotor bars faults) does not appear even when the fault is present; however, if the radial stray flux signal of the same figure is examined, the evolution of the harmonic *f*·(1–2·*s*) is evident. This highlights the importance of finding the readings of the different stray fluxes in the machine in order to obtain reliable diagnoses.

As has been shown by the results, the smart-sensor proposed here is capable of capturing and processing the different stray flux signals found in the induction motor frame: axial, radial, and the combination of both from a single triaxial stray flux sensor. This enables the proposal to reliably detect and diagnose diverse electromechanical faults.

## 5. Conclusions

This work proposes a new smart-sensor for online detection of individual and combined electromechanical faults in induction motors by analyzing the signals captured by a novel triaxial stray flux sensor, which results in a high portability. The smart-sensor has proven its capabilities to automatically and effectively diagnose individually, as well as the combination of two of the most common electromechanical faults that usually occur in induction motors: misalignment and broken rotor bars with high reliability, as the proposed intelligent algorithm relies on the transient analyses of both axial and radial stray flux components.

The smart-sensor presented here uses, as a primary sensor, an array of three hall-effect sensors located in perpendicular axes to each other, so that it is possible to simultaneously acquire, at a single position, the different stray flux components that contain information of great relevance for the diagnosis of failures in electric motors.

It was shown that, through the proposed triaxial sensor, it is possible to clearly visualize, by means of time–frequency maps, the appearance and evolution of characteristic patterns that arise in fault conditions during the start-up transient and that have been reported and justified in other works. In addition, the results are fully consistent with the theory, as they show that the harmonic *f·*(1–2*·s*), which is amplified by the presence of broken rotor bars, and is observed clearly and concisely by studying the radial stray flux, while the harmonic *s*·*f* (amplified by the presence of mechanical misalignments between the load and the motor and by the presence of broken rotor bars) makes its appearance with greater intensity in the time–frequency maps obtained by analyzing axial and the combination of the axial and radial stray flux. Considering this, the automated final diagnosis performed by the smart-sensor is based on data classification techniques such as linear discriminant analysis (LDA) and a feed-forward neural network (FFNN). These techniques together give the smart-sensor the ability to discern the weight of the different stray flux components on the final result.

The obtained results confirm the practical use of the smart-sensor as it is able to capture the different stray flux components found in electric motors and their evolution in a time–frequency map, characterize these fault components, perform signal processing in situ, show the results obtained through time–frequency maps to the end user, generate an automatic and timely diagnosis, and classify the different failures studied in this work, all in a very short time. Furthermore, the proposed smart-sensor is capable of adapting the study of other faults and it is planned to expand its capabilities as a future work by analyzing more test signals (obtained by the triaxial sensor) from the study of other faults such as static and dynamic eccentricities, bearing faults, and short circuits, among others.

## Figures and Tables

**Table 1:** Elapsed time for data acquisition and data processing performed by the smart-sensor. STFT, short time Fourier transform; LDA, linear discriminant analysis; FFNN, feed-forward neural network.

	Data Acquisition	Data Processing			
**Task**	Triaxial stray flux data acquisition	STFT (primary sensors 1, 2, and 3)	Statistical parameter extraction	LDA feature reduction	FFNN
**Elapsed time**	30 s	6.16 s	0.66 s	0.001 s	0.07 s

**Table 2:** Effectiveness of the proposed smart sensor on identifying the induction motor fault conditions studied here. HLT, healthy motor; BRB, broken rotor bar.

Induction Motor Condition	Effectiveness (%)
**HLT**	100
**MAL**	99.1
**1 BRB + MAL**	97
**2 BRB + MAL**	100
